# Dissection of the left main coronary artery after blunt thoracic trauma: Case report and literature review

**DOI:** 10.1186/1749-7922-5-21

**Published:** 2010-07-22

**Authors:** Mollie M James, Marnix Verhofste, Cass Franklin, Greg Beilman, Charles Goldman

**Affiliations:** 1 University of Minnesota, Department of Critical Care and Acute Care Surgery, Minneapolis, MN, USA; 2 Iowa Heart Center, Cardiothoracic Surgery Division, Des Moines, IA, USA; 3 Mercy Medical Center, Department of Trauma Services, Des Moines, IA, USA

## Abstract

Blunt chest trauma is commonly encountered by surgeons and is rarely associated with cardiac injuries. The incidence of cardiac injury is rare but can be rapidly fatal, requiring prompt recognition and treatment. We review the case of a 37 year-old male who was involved in a head-on motor vehicle collision at highway speed and was found to have an isolated left main coronary artery dissection. We then review the supporting literature for evaluation of blunt cardiac injuries and the treatment options for traumatic coronary dissection.

## Background

Blunt chest trauma is commonly encountered by trauma surgeons and has a variable clinical course. The spectrum of cardiac injuries ranges from mild cardiac contusion to cardiac failure or death. The diagnosis of blunt cardiac injury (BCI) can be challenging because chemical markers, nuclear studies and echocardiogram rarely correlate with the severity of injury. This article reviews a case of coronary artery dissection leading to acute ischemia, the diagnostic recommendations for evaluating patients at risk for BCI, and the therapeutic options for traumatic coronary artery dissection.

## Case Report

A 37 year-old white male presented as a trauma patient after a head-on motor vehicle collision at highway speed. The patient was the restrained driver; the driver of the other car was fatally injured at the scene. Primary survey revealed an intact airway. The patient was talking without stridor; breath sounds were equal bilaterally. Pulses were palpable in all extremities and there was no evidence of jugular venous distention. He was neurologically intact with a Glasgow Coma Score of 15. The patient complained of chest pressure and shortness of breath.

Initial vital signs were: systolic blood pressure 118 mmHg, pulse 99 beats per minute, respiratory rate 28 breaths per minute, and an oxygen saturation of 94% with supplemental oxygen at 2 liters per minute. He had a thoracic contusion consistent with a seatbelt sign and his sternum was tender to palpation. Physical findings also revealed a deformity of the left ankle. He had no history of medical problems or previous chest pain. Prior to the incident his only surgery was a knee arthroscopy. He had a 30 pack-year history of smoking and drank alcohol regularly.

**Figure 1:**
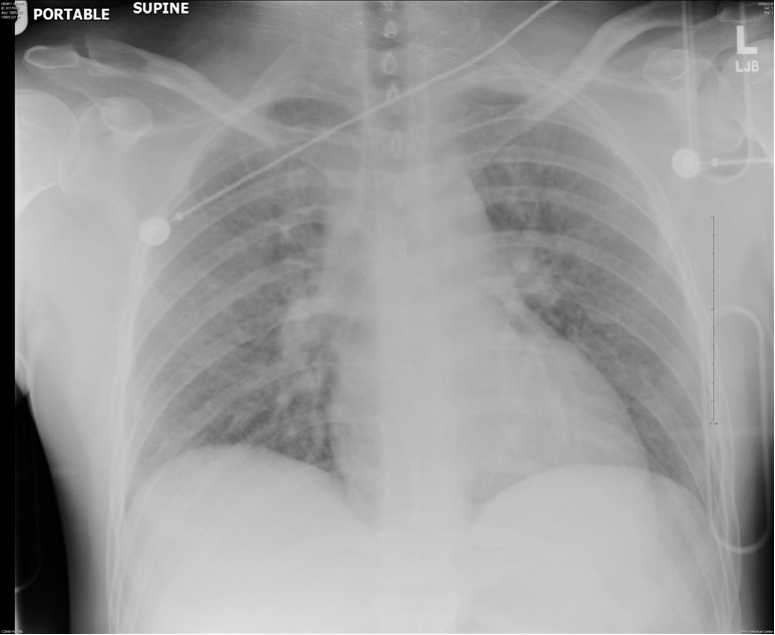
**The chest radiograph taken in the trauma bay does not demonstrate acute intrathoracic injury**.

**Figure 2:**
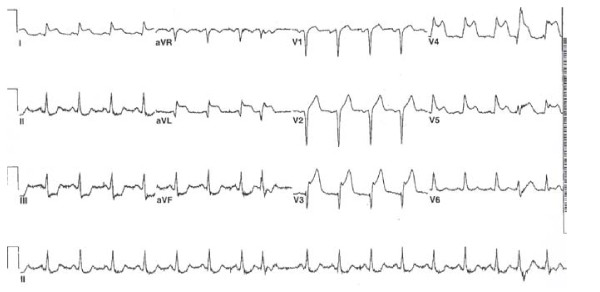
**The EKG demonstrates ST segment elevation in leads I, III, aVL, and aVF, as well as precordial leads V2-V5**. This suggests anterior wall ischemia and the patient underwent emergent coronary angiography.

**Figure 3:**
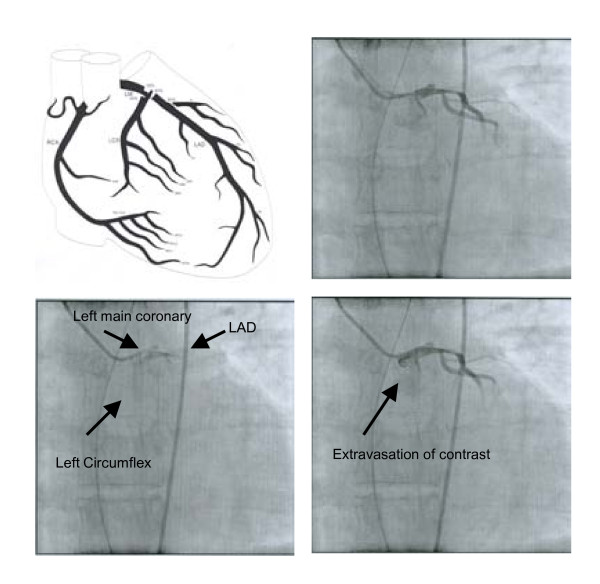
**The angiogram demonstrates a 50% diffuse stenosis of distal left main artery with left main dissection**. The LAD had 95% occlusion and 50% stenosis of the circumflex arteries. An intra-aortic balloon pump was placed and the patient was taken emergently to the operating room for coronary bypass.

The trauma evaluation was completed, including cervical spine, chest and pelvis radiographs, and a trauma laboratory panel (chest radiograph, Figure 1). An electrocardiogram (Figure 2) demonstrated acute ST elevation in leads I, aVL, aVF, and V2-V5. Based on the EKG findings suggesting ischemia, cardiac enzymes were ordered and, when noted to be elevated, the decision was made to proceed with a coronary angiogram. His cardiac enzymes were elevated with a creatinine phosphokinase of 454 ng/mL (38-120 ng/mL) creatinine phosphokinase-MB 13 ng/mL (< 3 ng/mL), and troponin of 0.02 ng/mL (< 0.04). He was given aspirin, intravenous morphine and metoprolol until his pain subsided. He underwent an emergent coronary angiogram (Figure 3) that demonstrated dissection of the left main coronary artery.

After the angiogram, the patient was taken urgently for a coronary artery bypass graft. At surgery he underwent a triple bypass graft as follows: reverse saphenous vein graft to obtuse marginal 1 (OM-1), reverse saphenous vein graft to ramus, and left internal mammary artery to left anterior descending artery. Postoperatively, he was admitted to the Surgical/Trauma Intensive Care Unit with an intra-aortic balloon pump (IABP) to augment cardiac function. He required re-exploration the first post-operative night for bleeding. A small uncontrolled side branch on the vein graft to the obtuse marginal artery was bleeding; it was repaired with a single 7-0 Prolene stitch.

After re-operation, the ejection fraction remained low at 15% per post-operative echocardiogram. He continued to require the IABP and vasopressors to sustain cardiac function. On the third post-operative day the IABP was removed; two days later vasopressor support was discontinued. Due to extensive injuries, he was not extubated until the twelfth day in the ICU. Concomitant injuries included left talus and calcaneus fractures that were surgically repaired during his hospital stay. He was discharged home on the 19^th ^hospital day with an ejection fraction of 30-35%.

## Discussion

### Evaluation of suspected cardiac injuries

The Eastern Association for the Surgery of Trauma (EAST) has published a practice management guideline for patients with suspected BCI. A review of the literature supporting these recommendations can be found in table 1. Each patient with suspected cardiac injury should have an EKG upon arrival (Level I) [1]. Abnormal admission EKG should likely be followed with cardiac monitoring for 24 hours or until hemodynamically stable. Patients with normal EKG and no symptoms or other injuries can be discharged after a brief period of observation. This recommendation is supported by a review by Christiansen that showed over 80% of patients who developed a clinically significant arrhythmia had EKG changes on the initial study, suggesting that intake EKG can be considered a reasonably discriminating screening exam [2].

**Table 1:** Review of Myocardial Contusion Evaluation in Blunt Thoracic Trauma

*Author/Journal*	*Number of patients*	*Number of cardiac complications*	*Conclusions*
Baxter, et al. [19] Retrospective 6 year review of all patients with blunt chest trauma	280	35 patients with myocardial contusion (MCC) 7 complications 2 deaths	* Complications of MCC manifest within 12 hours. * Patients with suspected MCC should have cardiac monitor and enzyme monitoring for 24 hours or until hemodynamically and electrically stable. * Patients with known coronary artery disease should have monitoring until hemodynamically stable and a myocardial infarction is ruled out. * Echocardiogram is helpful to further evaluate MCC.

Biffl, et al. [3] Retrospective 4-year review of all patients with high-risk blunt chest trauma	359	107 MCC 14 dysrhythmias 3 cardiogenic shock with 2 deaths	* Cardiac enzymes (CPK, CKMB) have no useful role in the evaluation of patients with myocardial contusion. * Risk factors associated with complications from MCC include age > 55, abnormal admission EKG (except sinus tachycardia), absence of chest pain, head injury with GCS < 8, and pelvic fracture.

Cachecho, et al [20] Retrospective 6-year review of patients with blunt thoracic trauma	336	19	*Young patients with minor blunt thoracic trauma and minimally abnormal EKG do not benefit from cardiac monitoring. * Evaluation of MCC should not be pursued in hemodynamically stable patients.

Karalis, et al [21] 12-month prospective evaluation of patients admitted with blunt thoracic trauma	105	8	* Only patients who have complications from MCC benefit from echocardiogram. Transesophageal echo may be beneficial if thoracic trauma limits the quality of a trans-thoracic study.

Adams, et al. [22] 12-month prospective evaluation of patients with blunt thoracic trauma	44	2 acute myocardial infarctions	Cardiac troponin I accurately detects cardiac injury after blunt chest trauma.

Echocardiography should be reserved for patients who are hypotensive either on admission or during the initial observation period. It can be helpful in diagnosing apical thrombi, pericardial effusion and tamponade. Echocardiograms added little clinical information for patients who were normotensive. Radionuclide imaging studies are too sensitive and lack specificity in the setting of trauma, so are not helpful in the evaluation of blunt cardiac trauma.

The EAST guidelines recommend against following cardiac enzymes because they are not helpful in predicting complications from BCI [1]. A review by Biffl, et al evaluated the management of suspected cardiac injury at a Level-One trauma center in Denver. Screening creatinine phosphokinase or troponin levels were frequently elevated post-injury and did not correlate with clinically significant BCI [3]. They identified clinical risk factors for complications in BCI including age greater than 55, an abnormal EKG at admission, the absence of chest pain, a widened mediastinum on imaging, a head injury with a Glasgow coma score less than 8, and pelvic fractures. In both univariate and multivariate analysis, these factors were more predictive of complications from BCI than cardiac enzymes [3].

Guidelines are helpful in directing the evaluation when thoracic injuries are suspected. The recommendations from EAST support a limited evaluation for negative screening tests and asymptomatic patients. If the initial screening evaluation is positive the algorithm is redirected to evaluate more specific injury patterns. In our case, there was evidence of malperfusion of the myocardial tissue in the anterior distribution, so we integrated cardiac enzymes and angiography to diagnose the potential causes of ST segment changes on the initial EKG. Efficiently proceeding from a screening evaluation to a diagnostic evaluation allowed for rapid detection and treatment of the coronary dissection.

Many types of cardiac injuries have been described after blunt chest trauma. Arrhythmia, cardiac contusion, and acute myocardial infarction are among the more common injuries [4]. Older patients can have ischemia induced by hemorrhagic shock superimposed on underlying cardiac disease, rather than from direct cardiac injury. Less commonly encountered are coronary artery laceration, thrombosis, or intimal dissection [4]. Clinically the injuries can by asymptomatic, or may cause angina, hemodynamic instability, or commotio cordis, resulting in sudden death.

### Coronary Artery Dissection

Coronary artery dissections are most common in the left anterior descending artery (76%), right coronary artery (12%) and the circumflex (6%) [5]. Very few cases have been reported from blunt trauma such as waterskiing [4], contact sports such as basketball [6] and football [5], and high-speed impact such as motorcycle[7,8], or motor vehicle collisions [9-12]. Dissection of the left main coronary artery is among the most rare sequela of blunt chest trauma. One trauma-related left main coronary dissection was reported 3 days after a head-on motor vehicle collision at only 15 mph [13]. Cases that have been reported in the literature are listed in table 2.

**Table 2:** Review of reported coronary artery dissections, treatment strategies, and outcomes

*Author/Journal*	*Patient age/sex*	*Mechanism*	*Injury*	*Treatment*	*Outcome*
Redondo, et al [11] Am J Emerg Surg, 2009	45 yo F	Motor vehicle collision	LMCA-focal stenotic dissection; RCA dissection	Angioplasty and heparin	Death secondary to intra-abdominal hemorrhage

Goyal, et al. [12] Heart, 2009	47 yo M	Motor vehicle collision	LMCA extending to LAD dissection	Unknown (no thrombolytics)	unknown

Harada, et al. [8] Ann Thorac Surg, 2002	14 yo M	Motorcycle collision	LMCA dissection with left ventricular aneurysm	Supportive care with surgical patch angioplasty and anuerysmectomy, mitral valvuloplasty and tricuspid annuloplasty 3 weeks later	Discharge to home; doing well 4 years post-operatively

Cini, et al [15] Interact Cardiovasc Thorac Surg, 2008	43 yo F	Spontaneous	LMCA dissection	Surgical revascularization	Discharge home

Rogers, et al Clin Cardiol, 2007	37 yo F (post-partum)	Spontaneous	LMCA with LAD involvement	Surgical revascularization	Discharge home

Hazeleger, et al. [5] Circulation, 2001	29 yo M	Tackled in football 2 months prior to arrival	LAD dissection; OM dissection	Stent	Discharge home

Smayra, et al. [10] Am J Thorac Cardiovasc Surg, 2007	17 yo M	Unrestrained motor vehicle collision 1 month prior to symptoms	LAD dissection	Surgical revascularization	Discharge home

Korach, et al [9] Interact Cardiovasc Thorac Surg, 2008	40 yo M	Pedestrian struck by automobile	LAD dissection	Surgical revascularization	Discharge home

Leong & Brown [7] Emerg Med J, 2006	50 yo M	Motorcycle collision	LAD dissection	Bare metal stent + angioplasty after thrombosis	Discharge home

Boland, et al. [13] Chest, 1988	32 yo F	Motor vehicle collision at 15 mph 3 days prior to admission	LAD & LCx dissection	Surgical revascularization	Discharge home

Vogiatzis, et al. [16] Hellenic J Cardiol, 2010	31 yo F (pregnant)	Spontaneous	LCx dissection	Conservative treatment without revascularization	Discharge home

Greenberg, et al. [4] Chest, 1998	35 yo F	Water-skiing 2 days prior to arrival	Circumflex artery dissection with moderate occlusion	Angiogram without intervention	Death due to brain death secondary to Vfib arrest prior to emergency department arrival

De Macedo, et al. [17] J Invasive Cardiol, 2009	34 yo M	Spontaneous	RCA dissection	Stent, heparin, clopidogrel, tirofiban, aspirin	Discharge home

Hobelmann[6] Emerg Med J, 2006	32 yo M	Elbow to chest in basketball	RCA dissection	Eptifibitide and heparin, stent X2	Discharge home

Table 2 Abbreviations: LAD: left anterior descending artery; LCx: left circumflex artery; RCA: right coronary artery; LMCA: left main coronary artery; OM: obtuse marginal artery; Vfib: ventricular fibrillation

Other causes of dissection unrelated to trauma include spontaneous lesions and iatrogenic injuries from coronary angiography. Spontaneous dissections have a 4:1 predilection for women with 25-33% occurring during pregnancy or the peripartum period [14]. Spontaneous lesions are associated with three conditions: 1) pre-existing coronary artery disease; 2) hormonal factors, such as pregnancy or oral contraceptive use, as stated above [14-16]; and 3) patients with tissue fragility disorders (e.g., Marfan's or Ehler-Danlos syndromes) [17]. Mortality with spontaneous dissection can be up to 70%, based on post-mortem studies after sudden cardiac death [17].

Iatrogenic injuries are rare, occurring in 3-6/10,000 angiograms. They are most commonly seen as RCA injuries, and can be due wire passage or balloon inflation [18].

### Treatment of Coronary Artery Dissection

The approach to treatment of coronary artery lesions is variable and depends upon the mechanism, the co-morbidities of the patient, and degree of hemodynamic stability. Conservative management includes anticoagulation and observation if they are hemodynamically stable with minimal injuries. Thrombolytics can be administered to dissolve clot associated with an intimal injury, but are contraindicated in multiply injured patients. Revascularization can be achieved with percutaneous techniques or coronary bypass, and timing is dependent upon the clinical scenario.

Advancements in percutaneous interventions have prompted some to attempt revascularization using this method. Lesions in the LAD and RCA are highly amenable to stent placement [23]. Surgical revascularization is the preferred therapy in coronary lesions with the following characteristics: 1) unprotected isolated LMCA disease, 2) contraindication to anti-platelet therapy including aspirin, heparin, and clopidogrel, 3) history of known coagulopathy or bleeding diathesis, or 4) pregnant women [23]. The need for subsequent anti-platelet therapy following stent placement to assure patency limits the utility of these approaches in the multiply injured blunt trauma patient. Some of these patients are already coagulopathic and the addition of these agents can destabilize clots in solid organs leading to life-threatening hemorrhage, or propagate an intracerebral hemorrhage with grave clinical consequences.

In our patient the decision to proceed to coronary bypass was likely due to two factors. Most importantly, the dissection involved the left main coronary artery, which is preferentially treated surgically [23]. Secondly, our patient had a contraindication to percutaneous techniques because of his risk of bleeding. Our approach is supported by a number of successful cases already reported. Korach, Smayra, and Boland all report cases of motor vehicle collisions with resultant LAD coronary dissection that were successfully treated with surgical revascularization [9,10,13]. Harada had a similar success story, but the dissection was the left main coronary artery [8]. Redondo reported a mortality in the case of a 45 year-old female diagnosed with a left coronary artery dissection after a motor vehicle collision [11]. Attempts to treat with angioplasty and heparinization were complicated by fatal intra-abdominal hemorrhage.

Coronary dissection after blunt chest trauma has been successfully treated with a more conservative approach. Hobelmann reported the case of a 32 year-old male who suffered an RCA dissection after being elbowed in the chest during basketball [6]. The lesion was successfully treated with eptifibitide, heparin and stents. A focal right coronary artery lesion can be successfully stented, similar to the treatment of lesions in coronary artery disease [23]. Also, the risk of bleeding associated with the use of anticoagulation and anti-platelet agents was lower due to the isolated nature of the trauma. Hazeleger reported an LAD dissection 2 months after a tackle in football which was successfully treated with a stent [5]. Once again, left anterior descending artery lesions respond well to stent placement [23]. Also, the time interval from injury to diagnosis significantly reduces the risk of bleeding from anticoagulation necessary when stents are utilized.

## Conclusions

Blunt thoracic injury is commonly encountered in a trauma center, and a small fraction of those patients will present with blunt cardiac injuries. The goal of evaluation should be identifying patients with clinically relevant complications related to the cardiac injury and providing the appropriate level of care to meet patients' needs. We present a review of the diagnostic tools for evaluating blunt cardiac injury.

Recommendations include obtaining an initial EKG on all patients at risk for injury. If the EKG is abnormal, cardiac monitoring may be reasonable for 24 to 48 hours or until the patient is asymptomatic and hemodynamically stable. Echocardiograms should be reserved for patients presenting with hemodynamic instability and can be helpful in identifying tamponade, pericardial contusion, or apical thrombi. Additional means of testing, such as serial enzyme monitoring, have additional costs with limited clinical benefit.

Coronary artery dissection is a rare clinical condition, with variable causes including trauma, iatrogenic lesions from angiography, and spontaneous dissections. Despite the etiology of the dissection, treatment is dependent upon the location of the lesion. Patients with LMCA lesions or those with a high-risk of bleeding will likely need to undergo coronary bypass. Lesions isolated to the LAD or RCA, and with isolated trauma, can be treated with percutaneous techniques.

In our patient sustained a high-risk blunt chest trauma from a motor vehicle collision. An EKG was ordered to evaluate his symptoms, and the screening test initiated a diagnostic evaluation. Based on those findings, additional diagnostic tests--the cardiac enzymes and angiogram--were justified and provided rapid diagnosis of the coronary artery dissection. Prompt recognition, evaluation and treatment resulted in immediate surgical revascularization and discharge to home on hospital day 19.

## Competing interests

The authors declare that they have no competing interests.

## Authors' contributions

MJ is the primary author and reviewed the case and the literature. MV, CF provided case review details and editorial input. GB provided direction for the paper and editorial commentary. CG was involved in writing and editing the paper.

All authors have read and approved the final manuscript.
